# Integrated transcriptomics and metabolomics analysis provide insight into the resistance response of rice against brown planthopper

**DOI:** 10.3389/fpls.2023.1213257

**Published:** 2023-06-20

**Authors:** Shaojie Shi, Wenjun Zha, Xinying Yu, Yan Wu, Sanhe Li, Huashan Xu, Peide Li, Changyan Li, Kai Liu, Junxiao Chen, Guocai Yang, Zhijun Chen, Bian Wu, Bingliang Wan, Kai Liu, Lei Zhou, Aiqing You

**Affiliations:** ^1^ Laboratory of Crop Molecular Breeding, Ministry of Agriculture and Rural Affairs, Hubei Key Laboratory of Food Crop Germplasm and Genetic Improvement, Food Crops Institute, Hubei Academy of Agricultural Sciences, Wuhan, China; ^2^ Hubei Hongshan Laboratory, Wuhan, China

**Keywords:** rice, brown planthopper, plant-insect interaction, multi-omics analysis, *Bph30*

## Abstract

**Introduction:**

The brown planthopper (Nilaparvata lugens Stål, BPH) is one of the most economically significant pests of rice. The Bph30 gene has been successfully cloned and conferred rice with broad-spectrum resistance to BPH. However, the molecular mechanisms by which Bph30 enhances resistance to BPH remain poorly understood.

**Methods:**

Here, we conducted a transcriptomic and metabolomic analysis of Bph30-transgenic (BPH30T) and BPH-susceptible Nipponbare plants to elucidate the response of Bph30 to BPH infestation.

**Results:**

Transcriptomic analyses revealed that the pathway of plant hormone signal transduction enriched exclusively in Nipponbare, and the greatest number of differentially expressed genes (DEGs) were involved in indole 3-acetic acid (IAA) signal transduction. Analysis of differentially accumulated metabolites (DAMs) revealed that DAMs involved in the amino acids and derivatives category were down-regulated in BPH30T plants following BPH feeding, and the great majority of DAMs in flavonoids category displayed the trend of increasing in BPH30T plants; the opposite pattern was observed in Nipponbare plants. Combined transcriptomics and metabolomics analysis revealed that the pathways of amino acids biosynthesis, plant hormone signal transduction, phenylpropanoid biosynthesis and flavonoid biosynthesis were enriched. The content of IAA significantly decreased in BPH30T plants following BPH feeding, and the content of IAA remained unchanged in Nipponbare. The exogenous application of IAA weakened the BPH resistance conferred by Bph30.

**Discussion:**

Our results indicated that Bph30 might coordinate the movement of primary and secondary metabolites and hormones in plants via the shikimate pathway to enhance the resistance of rice to BPH. Our results have important reference significance for the resistance mechanisms analysis and the efficient utilization of major BPH-resistance genes.

## Introduction

Rice (*Oryza sativa* L.) is the staple food for over 50% of population in the world (Zhang et al., 2018; Tan et al., 2020). BPH is widespread in Asia, Australia and South Pacific Islands and feeds on cultivar and several wild rice varieties (Jing et al., 2017; Zheng et al., 2021). BPHs consume the phloem sap of rice plants specifically, and their feeding activity on susceptible plants leads to the yellowing, browning, and drying of rice plants, eventually resulting in a phenomenon described as ‘hopperburn’ in the rice paddy (Jing et al., 2017; Tan et al., 2020). BPH can also serve as a vector for the spread of various viruses of rice, such as rice stunt viruses (Cheng et al., 2013; Sarao et al., 2016). The BPH has become the most serious pest that endangers rice production (Tan et al., 2020).

The application of chemical pesticides is one of the main strategies that has been used to control BPH infestation during the past 50 years (Du et al., 2020). However, BPHs have evolved resistance to most insecticides; this, coupled with the environmental pollution associated with insecticide use, has resulted in decreases in the use of chemical pesticides in several countries (Tamura et al., 2014; Du et al., 2020). Much effort is being made to develop the rice varieties resistant to BPH, which is considered an effective and environmentally benign method for the management of BPH (Du et al., 2020). To date, at least 41 resistance loci to brown planthopper have been discovered, and the BPH-resistance function of 17 genes has been experimentally confirmed (Muduli et al., 2021; Shi et al., 2021; Zhou et al., 2021). But, a lack of knowledge of the mechanisms by which these genes confer BPH resistance precludes their effective use for the breeding of rice varieties resistant to BPH. *Bph14* was the first cloned brown planthopper resistance gene in the world; this gene encodes a NLR protein. BPH14 enhances BPH resistance, through the reactive oxygen species (ROS) accumulation, salicylic acid (SA) signaling activation and the promotion of callose deposition in sieve tubes. (Du et al., 2009; Hu et al., 2017). BPH-resistance protein BPH6 mediates the activation of the SA, jasmonic acid (JA) and cytokinin (CK) signaling and promotes the deposition of callose in the phloem following infection of *Bph6*-containing plants with BPHs (Guo et al., 2018). Recently, a study showed that BPH6 interacts with the exocyst subunit OsEXO70H3 and *S*-adenosylmethionine synthase-like protein (SAMSL), promotes SAMSL secretion, increases the content of lignin in the cell wall, and enhances BPH resistance (Wu et al., 2022). The results of the above studies suggest that the BPH resistance conferred by several major BPH-resistance genes in rice is mediated by various resistance pathways.

High-throughput omics approaches have made substantial contributions to the complex mechanisms analysis of key BPH-resistance genes. High-throughput RNA sequencing have revealed that the BPH resistance conferred by *Bph15* is related to plant hormones, transcription factors, receptor kinase, protein post translational modifications, mitogen-activated protein kinase cascades, Ca^2+^ signaling and pathogenesis-related protein. (Lv et al., 2014). A subsequent microRNA sequencing analysis has revealed 23 differentially expressed miRNAs between 9311 and 9311-*Bph15*-NIL following BPH feeding, which targeted to those genes involved in abiotic and biotic stimuli, regulation of plant hormones, cellulose biosynthesis, amino acid synthesis, and protein folding, that mainly related to BPH resistance. (Wu et al., 2017). A total of 24 DEGs have been identified in both the *Bph6*-transgenic plants and Nipponbare plants following BPH infestation, whose expression trends in the two materials were opposite and considered BPH resistance-related (Tan et al., 2020). Multiple differentially accumulated metabolites (DAMs) can be detected in plants using metabolomic approaches (Alamgir et al., 2016; Peng et al., 2016; Liu et al., 2017). DAMs between TN1 rice plants, which are susceptible to BPH, and *Bph15*-containing plants, are involved in the shikimate pathway, and these metabolites are exclusively enriched in *Bph15*-containing plants (Peng et al., 2016). Thus, the *Bph15* gene might enhance BPH resistance through its effects on the shikimate pathway (Peng et al., 2016). Analysis of lipid profiles has revealed that BPH feeding activity promotes phytol and wax metabolism in *Bph6*-transgenic plants, which indicates that these metabolites play key roles in the responses of *Bph6* to BPH infestation (Zhang et al., 2018). Most previous studies examining the mechanisms underlying the BPH resistance conferred by key BPH-resistance genes have used omics approaches to characterize differences in gene expression or changes in metabolites in rice materials following BPH feeding. By contrast, few studies have used combined transcriptome and metabolome analyses to systematically study the mechanisms by which key BPH-resistance genes confer BPH resistance.

Primary and secondary (or specialized) metabolites, as well as plant hormones were the three main groups of metabolites in the plant kingdom (Erb and Kliebenstein, 2020). Such as amino acids, which belong to primary metabolites, are required for plant growth. Meanwhile primary metabolites are important nutrient sources for invaders as well; these metabolites are thus involved in the responses of plants to biotic stress (Abood and Lösel, 2003; Solomon et al., 2003; Jobic et al., 2007). Secondary metabolites mediate plant-environment interactions (Hartmann, 2007). For example, the production of serotonin is induced by BPH infestation, and the inhibition of serotonin production improved rice’s ability to resist BPH (Lu et al., 2018). Flavonoids are the most studied secondary metabolites of crops that are toxic to insects, such as rutin, kaempferol, tricin and quercetin can effectively prevent pests damage to plants (Zhang et al., 2017; Aboshi et al., 2018; Su et al., 2021). Phytohormones also play key roles in plants counteract to external stress. In the early immune response, a complex hormone signaling network is induced by infections caused by invaders (Chen et al., 2021). JA and SA are the two major immune-related phytohormones in plants response to biotic stress (Zhao et al., 2016; Zhang and Li, 2019; He et al., 2020). Other phytohormones, such as CK, ethylene and abscisic acid (ABA) were also reported to be involved in plant defense (Erb et al., 2009; Dervinis et al., 2010; Hu et al., 2011). Primary metabolism, secondary metabolism, and plant hormones are closely linked in plants, and they comprise an efficient defense system for coping with environmental stress (Erb and Kliebenstein, 2020; Chen et al., 2021). However, most previous studies examining the mechanisms of resistance conferred by major BPH-resistance genes have focused on only one aspect of this defense system (Zhao et al., 2016; Lu et al., 2018; Zhang et al., 2018).

*Bph30* gene has been cloned from the BPH-resistance rice variety AC-1613 by map-based cloning. BPH30 protein contains two leucine-rich domains (LRDs), enhances the sclerenchyma stiffer and thicker, granting rice resistance to five biotypes of BPH and WBPH (white-backed planthopper). But, the molecular mechanisms by which *Bph30* confers BPH resistance for rice were poorly cleared. Here, we conducted a joint analysis of transcriptome and metabolome to characterize the response of *Bph30* to BPH infestation. Our results revealed that the shikimate pathway, amino acid biosynthesis, phenylpropanoid metabolism, flavonoids, lignin and indole 3-acetic acid (IAA) biosynthesis, and IAA signal transduction were involved in the mechanism of *Bph30*-mediated BPH resistance. We also validated the negative role of IAA in *Bph30*-mediated resistance to brown planthopper. Our results provide a comprehensive overview of the sophisticated mechanism in *Bph30* responses to BPH infestation and will aid the utilization of major BPH-resistance genes in breeding programs aimed at the development of BPH-resistant rice varieties.

## Materials and methods

### Plants and insects

In this study, we mainly used two types of rice materials. Nipponbare was the BPH-susceptible model variety and the genetically modified background material. BPH30T was *Bph30*-transgenic plants, which was constructed by transforming the *Bph30* genome that cloned from BPH-resistance rice AC-1613 into the Nipponbare background, as previous description (Shi et al., 2021).

The brown planthopper used in this study was a population captured from the rice field in Wuhan, China, and bred on BPH-susceptible TN1 variety for multiple generations. The feeding conditions are 26°C ± 0.5°C, 16-h-light/8-h-dark cycle in Wuhan University (Jing et al., 2012).

### BPH resistance evaluation of rice

Evaluation method for phenotypic resistance of rice varieties to brown planthopper was described as previously (Huang et al., 2001; Du et al., 2009). In brief, approximately 15-20 seeds with consistent germination were sown in a circular plastic cup (10-cm-diameter). After 2 weeks of growth, approximately three-leaf stage, infested with BPH according to the standard of 10 insects per seedling. Observed the phenotype of seedlings every day until the susceptible Nipponbare control died (a resistance assay score of 9) or the experimental materials shown the damaged phenotype, each seedling was assigned a resistance score, as described previously (Huang et al., 2001). Three replications were conducted.

### Measurement of BPH honeydew excretion and weight gain

The measuring method of BPH honeydew excretion and weight gain was as described previously (Shi et al., 2021). The rice seedlings used in this assay were four-leaf stage. The BPHs used in this assay were the newly emerged short winged female insects. Firstly, the weight of BPHs and parafilm sachets were measured. Then, fixed the pre-weighed parafilm sachet to the base of the rice seedlings, and enclosed one known weight brown planthopper into one parafilm sachet for free feeding for 48 h. Finally, removed the brown planthoppers and the parafilm sachets, and measured the weight of the brown planthopper and the parafilm sachets after feeding. The difference in BPHs weight between before and after feeding was weight gain, and the difference in weight of parafilm sachets before and after BPH feeding was honeydew excretion. 15 replicates were used for analysis in each experiment.

### RNA isolation and genes expression analysis

The outermost leaf sheaths of rice seedlings were peeled off and ground into powder in liquid nitrogen immediately. The total RNA was extracted from the tissue powder using TRIzol reagent. Reverse transcription of RNA into cDNA using PrimeScrip RT Reagent Kit (Takara, RR047Q). Application of SYBR Green PCR Master Mix (Applied Biosystems) on CFX96 Real-Time System (Bio-Rad) for gene expression detection. Each group of samples contained 3 biological replicates. *OsActin1* was selected as the internal reference gene and calculated the expression level of genes using 2^-ΔΔC(t)^ method. The rice seedlings used in this experiment were at four-leaf stage. Primer sequences used in this section were listed in **Supplementary Table 13**.

### Construction of the cDNA library and RNA sequencing

The leaf sheaths of Nipponbare and BPH30T at four-leaf stage were used for RNA-sequence. The method of sample preparation: each seedling was fed with 10 brown planthopper nymphs for 48 h as the experimental groups and seedlings not fed by brown planthopper as control groups; the outermost leaf sheaths of seedlings in both the experimental and control groups were simultaneously peeled off and stored in dry ice for subsequent processing. Each treatment contained three biological replicates. The isolation of total RNA, generation of sequencing libraries and RNA sequencing were conducted by Shanghai Personal Biotechnology Co., Ltd.

### RNA mapping and differential expression analysis

The low-quality and adaptor sequences were filtered out from raw reads using Trimmomatic and the clean reads were obtained. Then aligned the clean reads to the reference genome Os-Nipponbare version IRGSP-1.0 (annotation version 2022-09-01, https://rapdb.dna.affrc.go.jp/download/irgsp1.html) with Hisat2. The expression level of genes was measured by transcripts per Million (TPM). DESeq2 R package were used for the screening of differentially expressed genes (DEGs), the threshold of screening was |log_2_ (Fold Change)|≥1 and False discovery rate (FDR) ≤ 0.05.

### Function analysis of DEGs

DEGs obtained through the above method, were compared to the whole-genome background with hypergeometric test for Gene Ontology (GO) and Kyoto Encyclopedia of Genes and Genomes (KEGG) functional analysis. The hypergeometric test was corrected by Benjamini and Hochberg false discovery rate and the significance threshold is 0.05.

### Metabolites analysis

The samples used in metabolomic analysis were the leaf sheaths of Nipponbare and BPH30T at four-leaf stage. The outmost leaf sheath was quickly peeled off and stored in dry ice and sent to Wuhan Maiwei Biotechnology Company for samples pretreatment, metabolites extraction, detection, qualitative, quantitative, and differentially accumulated metabolites (DAMs) analysis. The ultra performance liquid chromatography-tandem mass spectrometry (UPLC-MS/MS) was used to detect the extracted metabolites. The threshold of variable importance in projection (VIP) ≥ 1 and |log_2_ (Fold Change)| ≥ 1 was used for detecting DAMs in two-group analysis.

### PCA computational method

In this study, the statistics function prcomp within R (www.r-project.org) was used to conduct principal component analysis (PCA). Before PCA, the data was unit variance scaled.

### Metabolites annotation and enrichment analysis

Metabolites identified were annotated by comparing with KEGG compound database (http://www.kegg.jp/kegg/compound/). The metabolic pathways that mapped by annotated metabolites were screened out from KEGG pathway database (http://www.kegg.jp/kegg/pathway.html). Then, the metabolite sets enrichment analysis on metabolic pathways labeled with significantly different metabolites were performed using *p* ≤ 0.05 as the significance threshold.

### IAA content measurements

The IAA contents of the outermost leaf sheaths of rice seedlings at four-leaf stage were measured. Firstly, the leaf sheaths were peeled off and ground into powder in liquid nitrogen and IAA that in 0.2 g sample powder, was extracted by acetonitrile solution. Then, add 50 mg C18 filler to the acetonitrile solution that dissolved IAA, mix well, centrifuge, take the supernatant, and blow dry with nitrogen gas. Finally, add 200 μl methanol to the sediment for complete dissolution, filtered with 0.22 μm organic phase filter membrane, and the IAA content was detected by HPLC-MS/MS at Nanjing Webiolotech Biotechnology Co., Ltd.

### IAA treatment

Prepare the Indole 3-acetic acid (IAA, I2886, Sigma-Aldrich) solution with a concentration of 1µm. Then the BPH30T seedlings were sprayed with IAA solution. After two hours, BPH were released on the seedlings.

### Data analysis

All data in this study were statistically analyzed using one-way analysis of variance with PASW Statistics version 18.0.

## Results

### Evaluation of the resistance of *Bph30*-transgenic (BPH30T) rice plants to BPH infestation

**Figure 1:**
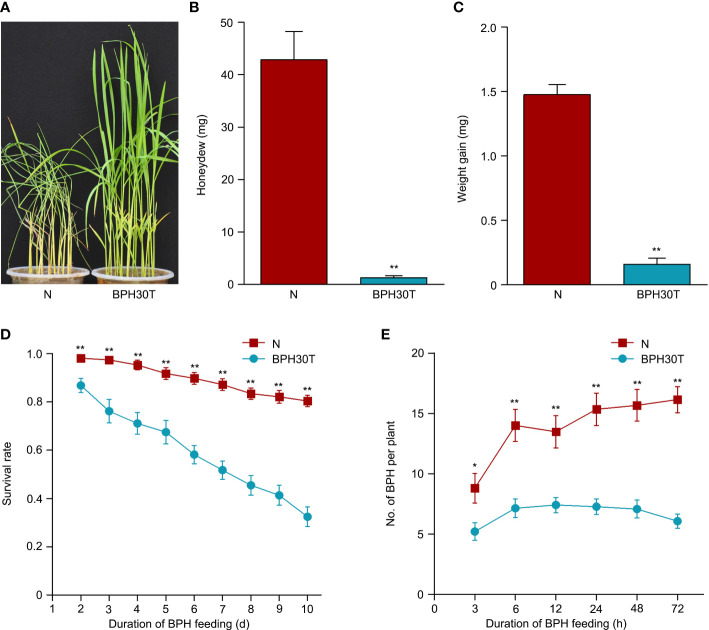
BPH resistance evaluation of BPH30T and Nipponbare plants. **(A)** Seedling test identified the phenotype of resistance to BPH of *Bph30*-transgenic plants (BPH30T) and Nipponbare (N). **(B)** The secretion of honeydew by BPH after feeding on BPH30T and N for 48 h. **(C)** BPH weight gain after feeding on BPH30T and N for 48 h. **(D)** Survival rate of BPH feeding on BPH30T and N. **(E)** Selectivity of brown planthopper in BPH30T and N. In **(B–E)**, data represent the means ± SEM. Asterisks indicate significant differences (*represent *p* < 0.05, **represent *p* < 0.01).

Previous study has indicated that *Bph30* has strong resistance to brown planthopper (Shi et al., 2021). In this study, the phenotype of *Bph30* was verified using *Bph30*-transgenic plants (BPH30T) that with Nipponbare genetic background and contain the genomic fragment of *Bph30* with its native promoter. In the bulk seedling test, when the BPH-susceptible variety Nipponbare all die caused by brown planthopper feeding, the BPH30T plants grow well (**Figure 1A**). Honeydew excretion and weight gain of BPHs that fed on Nipponbare for 48 h were significantly greater than that fed on BPH30T plants (**Figures 1B, C**). The survival rate of BPHs after 2 days was significantly higher when they fed on Nipponbare plants than that fed on BPH30T plants (**Figure 1D**). In addition, host-choice tests revealed that a significantly higher number of BPHs settled on Nipponbare than that settled on BPH30T plants from 3-72 h following the release of BPHs (**Figure 1E**). These findings suggested that BPH30T plants were highly resistant to BPH.

Previous study has shown that BPH30 enhances the strength of cell wall by promoting the cellulose and hemicellulose biosynthesis (Shi et al., 2021). Cellulose synthesis-related genes, *BC15* (*Os09g0494200*, brittle culm 15), *CESA7* (*Os10g0467800*, cellulose synthase 7), *CSLF4* (*Os07g0553300*, cellulose synthase-like F 4) and hemicellulose synthesis-related gene, *IRX9* (*Os07g0694400*, irregular xylem 9), were selected for expression analysis in Nipponbare and BPH30T plants. Our findings indicated that the expression of cellulose and hemicellulose synthesis-related genes was significantly lower in Nipponbare, before and after BPH feeding, than that in BPH30T plants (**Supplementary Figure 1**).

### Transcriptomic analysis of Nipponbare and BPH30T response to BPH feeding activity

To clarify the mechanism by which *Bph30* confers BPH resistance, RNA sequencing analysis was conducted using leaf sheaths from Nipponbare and BPH30T plants before (S0 and R0) and after BPH feeding for 48 h (S48 and R48). The total number of raw reads generated ranged from 20,579,084 to 28,035,649 for each library. The clean reads ranged from 19,514,119 to 26,954,105, with an average Q30 score of 92.10% to 95.33%, after filtering out adapters, low-quality and uncertain reads. Then, the clean reads were aligned to the genome of Nipponbare (https://rapdb.dna.affrc.go.jp/download/irgsp1.html). The matching rate ranged from 92.47% to 98.63% for Nipponbare and BPH30T plants (**Table 1**). Correlation analysis between intra-group samples revealed high correlations among the three replicates within groups, suggesting that the repeatability of the biological replicates and the accuracy of the RNA-seq data were high (**Supplementary Figure 2**).

**Table 1:** Statistics of sequencing reads and alignment to the reference genome.

Sample	Raw reads	Clean reads	Q30 (%)	Percentage of alignment (%)
S0-1	26,175,802	24,656,295	93.69	97.35
S0-2	23,155,745	21,869,050	94.12	97.64
S0-3	23,612,057	22,159,036	93.10	97.26
S48-1	20,579,084	19,514,119	94.53	92.47
S48-2	22,683,817	21,301,586	93.30	97.65
S48-3	22,087,338	20,685,803	92.10	92.32
R0-1	23,000,390	21,694,021	94.60	97.77
R0-2	21,083,323	19,928,730	94.19	97.78
R0-3	23,379,633	21,994,210	93.81	97.88
R48-1	22,866,552	21,546,357	93.58	98.49
R48-2	25,583,036	24,136,491	93.93	98.44
R48-3	28,035,649	26,954,105	95.33	98.63

S0, R0, S48 and R48 represent the Nipponbare (S) and BPH30T (R) before (S0 and R0) and after BPH feeding for 48 h (S48 and R48) respectively. 1, 2, 3 represent the three biological repeats.

**Figure 2:**
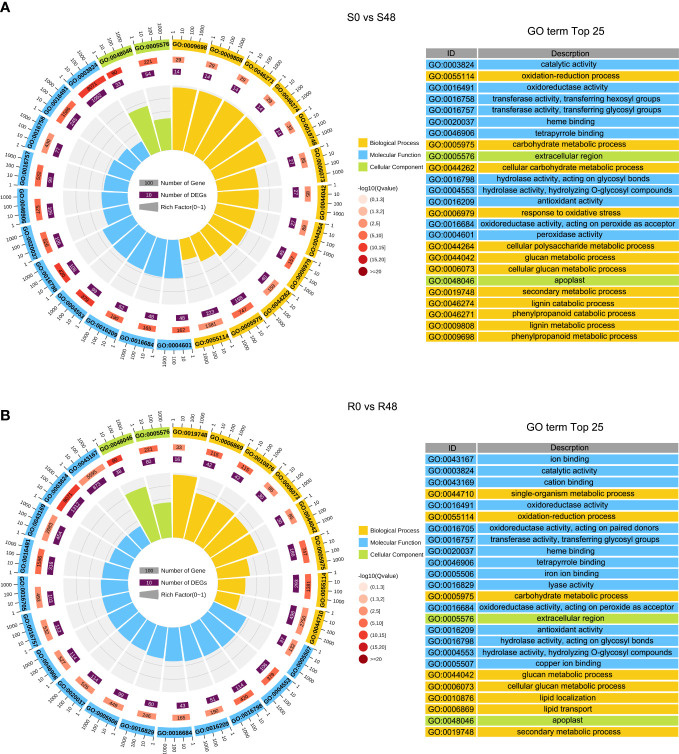
GO (Gene Ontology) enrichment analysis of DEGs detected from S0 vs S48 and R0 vs R48 groups. **(A, B)** GO analysis of DEGs in S0 vs S48 **(A)** and R0 vs R48 **(B)**. S0, R0, S48 and R48 represent the Nipponbare (S) and BPH30T (R) before (S0 and R0) and after BPH feeding for 48 h (S48 and R48) respectively. Outer ring, GO terms; second ring, the number of genes enriched into each GO term in the whole genome; third ring, number of genes detected in this study enriched in each GO term; fourth ring, rich factor. The spacing between two lines in the background represents 0.1. .

**Figure 3:**
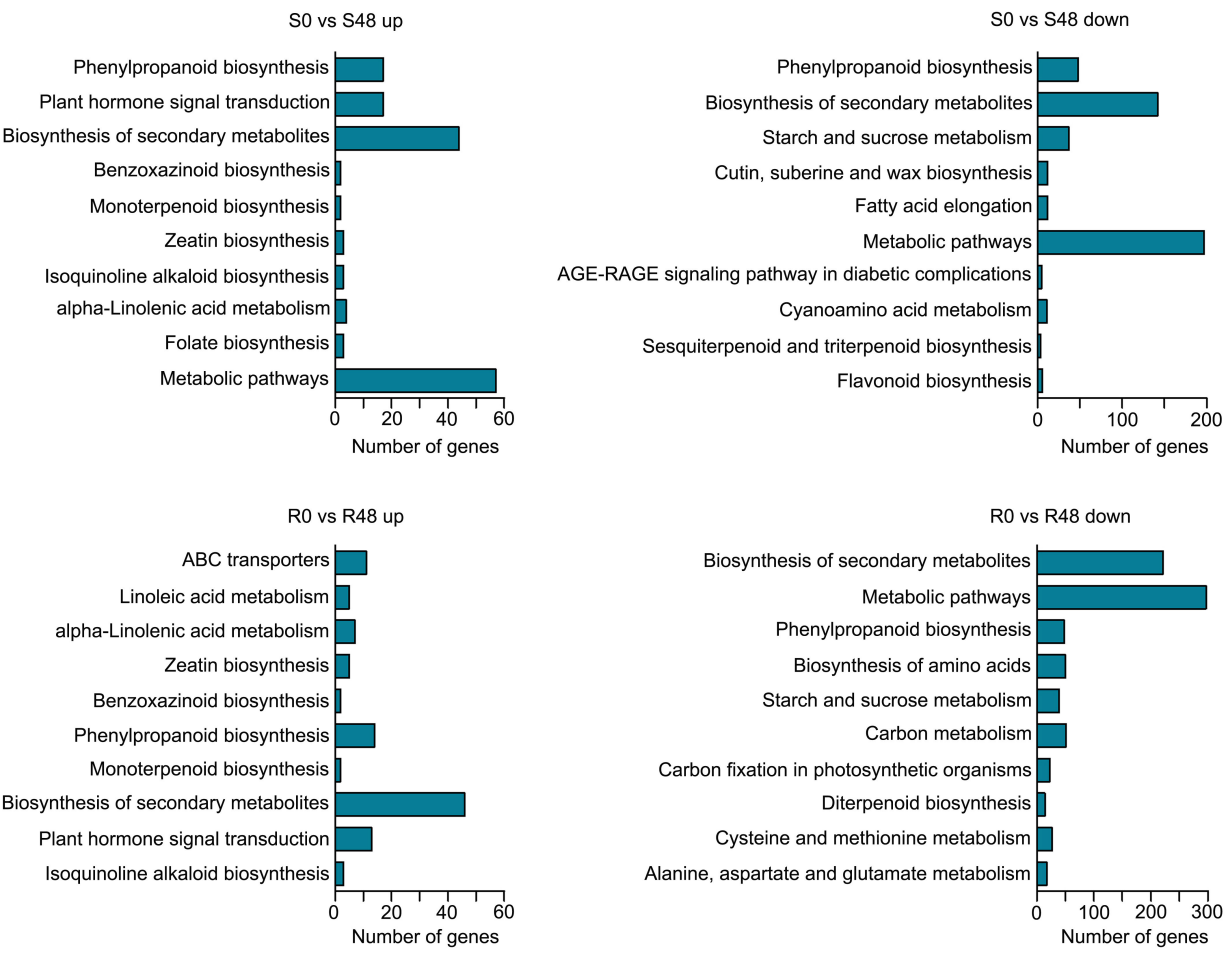
KEGG functional analysis of DEGs from the two varieties after BPH feeding compared with unfed control. Enriched KEGG pathways of up-regulated DEGs in Nipponbare and BPH30T (S0 vs S48 up, R0 vs R48 up) and down-regulated DEGs in Nipponbare and BPH30T after BPH feeding for 48 h (S0 vs S48 down, R0 vs R48 down).

To characterize transcriptional-level changes in the two cultivars induced by BPH feeding activity, we conducted an analysis of DEGs, which were identified using the following criteria: |log_2_ (Fold Change)| ≥ 1 and FDR ≤ 0.05. There were 3,407 DEGs identified in the S0 vs S48 comparison group, including 912 DEGs that up-regulated expression in S48 and 2,495 DEGs that down-regulated in S48 (**Supplementary Table 1**; **Supplementary Figure 3**). A total of 4,313 DEGs were detected in the R0 vs R48 comparison group, including 1,264 DEGs that up-regulated expression in R48 and 3,049 DEGs down-regulated in R48 (**Supplementary Table 2**; **Supplementary Figure 3**). To elucidate the molecular mechanisms of the DEGs, we carried out the Gene Ontology (GO) and Kyoto Encyclopedia of Genes and Genomes (KEGG) analyses using those DEGs. The results of GO enrichment analysis revealed that the DEGs identified in Nipponbare plants following BPH feeding were mainly enriched in the following GO terms: cellular carbohydrate metabolic process, response to oxidative stress, cellular polysaccharide metabolic process, lignin catabolic and metabolic process, phenylpropanoid catabolic process, phenylpropanoid metabolic process, and so on in the biological process, transferase and peroxidase activity in molecular function and apoplast, extracellular region in cellular component category. However, the DEGs identified in BPH30T plants following BPH infestation were mainly enriched in the following GO terms: single-organism metabolic process, lipid localization, and lipid transport in the biological process; ion and cation binding, oxidoreductase and lyase activity in the molecular function; apoplast, extracellular region and so on in cellular component category (**Figure 2**; **Supplementary Tables 3**, **4**). KEGG pathway analysis revealed that the DEGs in the two rice varieties following BPH feeding were enriched in phytohormone signal transduction; benzoxazinoid, suberine, cutin, phenylpropanoid and wax biosynthesis; starch, sucrose and cyanoamino acid metabolism. However, the expression of DEGs enriched in biosynthesis of flavonoids were down-regulated in Nipponbare, as well as the DEGs involved in the sesquiterpenoid and triterpenoid biosynthesis, which were involved in the mechanism of indirect defense against BPHs in rice plants, were also down-regulated in Nipponbare (Kamolsukyeunyong et al., 2021). In BPH30T plants, we found that the DEGs enriched in the biosynthesis pathway of amino acid and carbon fixation, which are the sources of the most important nutrients for BPHs, was down-regulated (**Figure 3**; **Supplementary Table 5**).

These findings indicated that the responses of Nipponbare plants and BPH30T plants to BPH feeding activity differed. We speculate that the synthesis of toxic and defensive secondary metabolites is reduced in Nipponbare plants following BPH feeding activity, which facilitated the maintenance of normal primary metabolic processes, as well as the success of infection by BPHs. However, the synthesis of primary metabolites is reduced in BPH30T plants, which facilitates the synthesis of toxic and defensive secondary metabolites and impedes BPH infection.

### Identification of DEGs involved in BPH resistance

**Figure 4:**
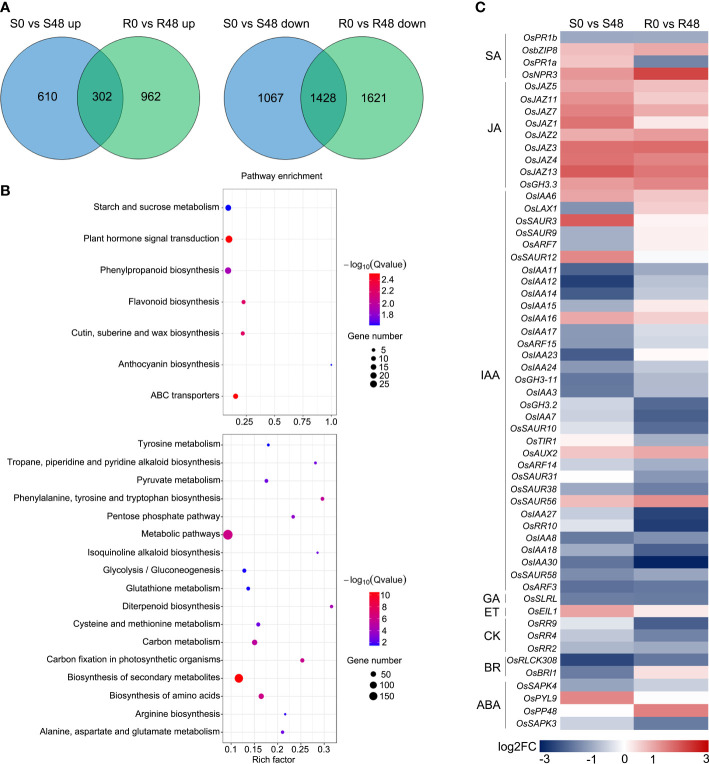
Unique DEGs identified from the two rice varieties after BPH infestation. **(A)** Venn diagrams illustrated the unique DEGs in two rice varieties before and after fed by brown planthopper. **(B)** KEGG pathway analysis of the unique DEGs from Nipponbare (up) and BPH30T (down) fed by BPH for 48 h. **(C)** Heat map displayed the unique DEGs related to plant hormone signal transduction in two comparison groups.

Venn diagrams were made to identify unique and overlapped DEGs in Nipponbare and BPH30T plants to clarify changes in transcription level. A total of 1,677 DEGs, which including 1,067 DEGs were up-regulated and 610 DEGs were down-regulated expression, were detected in Nipponbare following BPH feeding. 2,583 DEGs were identified in BPH30T plants following BPH infestation activity, which contained 1,621 DEGs that were up-regulated and 962 DEGs down-regulated. There were 302 up-regulated and 1,248 down-regulated DEGs overlapped in two varieties during BPH infestation (**Figure 4A**). In the overlapped DEGs sets, we found the expression of several biotic stress related genes exhibited similar changing trend in response to BPH feeding. Such as WRKY transcription factors, *OsWRKY45*, *OsWRKY62*, *OsWRKY65*, *OsWRKY104*, *OsWRKY114*; the gene encoding E3 ligases, *OsBBI1*; NPR1-like gene, *OsNPR3*; all of which were reported involved in plant resistance (Peng et al., 2008; Li et al., 2011; Huangfu et al., 2016; Jiang et al., 2020; Son et al., 2020; Chen et al., 2022; Son et al., 2022). Those findings indicated there may be a common foundational defense mechanism in resistant and susceptible rice varieties to response brown planthopper infestation. But, unique DEGs in Nipponbare and BPH30T plants might affect susceptibility and resistance of *Bph30* to BPHs. KEGG analysis showed these unique DEGs were enriched in different pathways in Nipponbare and BPH30T plants (**Supplementary Tables 1**, **2**). In Nipponbare plants, the unique DEGs were mainly enriched in the following KEGG pathways: flavonoid, phenylpropanoid, cutin, suberine and wax biosynthesis; ABC transporters and plant hormone signal transduction. In BPH30T plants, the unique DEGs were mainly enriched in the metabolic pathways; biosynthesis of amino acids, secondary metabolites, phenylalanine, tyrosine and tryptophan; carbon fixation in photosynthetic organisms (**Figure 4B**).

Plant hormone signals play key roles in plant-insect interaction, and the pathway of plant hormone signal transduction was only enriched in Nipponbare. To characterize differences in plant hormone signal transduction between Nipponbare and BPH30T plants following BPH feeding activity, a heat map was made using DEGs involved in this pathway. The greatest number of genes were involved in IAA signal transduction (**Figure 4C**). Most of these DEGs were down-regulated in both Nipponbare and BPH30T, but the extent to which these genes were down-regulated differed in Nipponbare and BPH30T plants, indicating that IAA might play a role in mediating BPH resistance conferred by *Bph30*.

### Metabolomic analysis of rice during the response to BPH feeding activity

**Figure 5:**
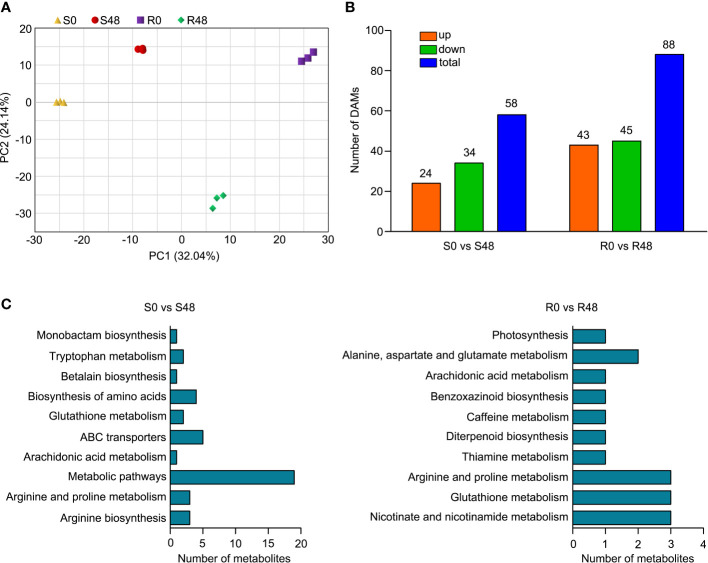
Differentially accumulated metabolites (DAMs) identified from the two rice varieties after BPH feeding for 48 h. **(A)** Principal component analysis (PCA) plots of total detected metabolites of S0, S48, R0 and R48. **(B)** The numbers of total, up- and down-regulated DAMs in two comparison groups. **(C)** KEGG pathway analysis of the DAMs in Nipponbare and BPH30T fed by BPH for 48 h.

To further reveal the response of *Bph30* to brown planthopper feeding at the molecular level. We extracted the total metabolites from Nipponbare and BPH30T plants leaf sheaths before BPH feeding and after BPH infestation for 48 h, and conducted the analysis of metabolite changes in the two rice varieties using UPLC-MS/MS. 1,198 metabolites from 11 different classes were detected (**Supplementary Table 6**). Coumaroylquinic acid, neochlorogenic acid, eudesmic acid, D-glucopyranoside, D-Glucurono-6,3-lactone, erythorbic acid, 2,4-Dihydroxybenzaldehyde, 4-Hydroxybenzoylmalic acid, apigenin-7-O-glucoside and 3,4-Dimethoxycinnamic acid were the first 10 metabolites that had the largest positive or negative loading values for PC1 in the principal component analysis (PCA). The results of PCA also illustrated differences among groups, biological replicates from the same group were clustered (**Figure 5A**), suggesting that the metabolite data were reproducible and suitable for subsequent data analyses.

There were 58 DAMs that were detected in Nipponbare plants, in the S0 vs S48 comparison group using |log_2_ (Fold Change)| ≥ 1 and VIP ≥ 1 as the filtering criteria; 24 and 34 of these DAMs were up-regulated and down-regulated, respectively (**Figure 5B**; **Supplementary Table 7**). A total of 88 DAMs were detected in BPH30T plants in the R0 vs R48 comparison group, and 43 and 45 of these DAMs were up-regulated and down-regulated, respectively (**Figure 5B**; **Supplementary Table 8**). To clarify the functions of DAMs detected from both Nipponbare and BPH30T plants following BPH infestation, we carried out the KEGG enrichment analysis on those DAMs. The DAMs that detected from Nipponbare, were significantly enriched in the KEGG functional pathways of arginine biosynthesis, arachidonic acid metabolism, ABC transporters, glutathione metabolism, biosynthesis of amino acids, and betalain biosynthesis. In BPH30T plants, DAMs were enriched in several KEGG pathways, including glutathione metabolism, nicotinate and nicotinamide metabolism, arginine and proline metabolism, benzoxazinoid biosynthesis, caffeine metabolism, and diterpenoid biosynthesis (**Figure 5C**; **Supplementary Tables 9**, **10**). These findings indicated that metabolites including primary and secondary metabolites altered greatly in both BPH-susceptible Nipponbare and BPH-resistance BPH30T plants, however, the number of DAMs and the enriched pathways identified in Nipponbare and BPH30T plants were distinct, suggesting that their responses to BPH infestation differed.

### Identification of metabolites that mediate BPH resistance

**Figure 6:**
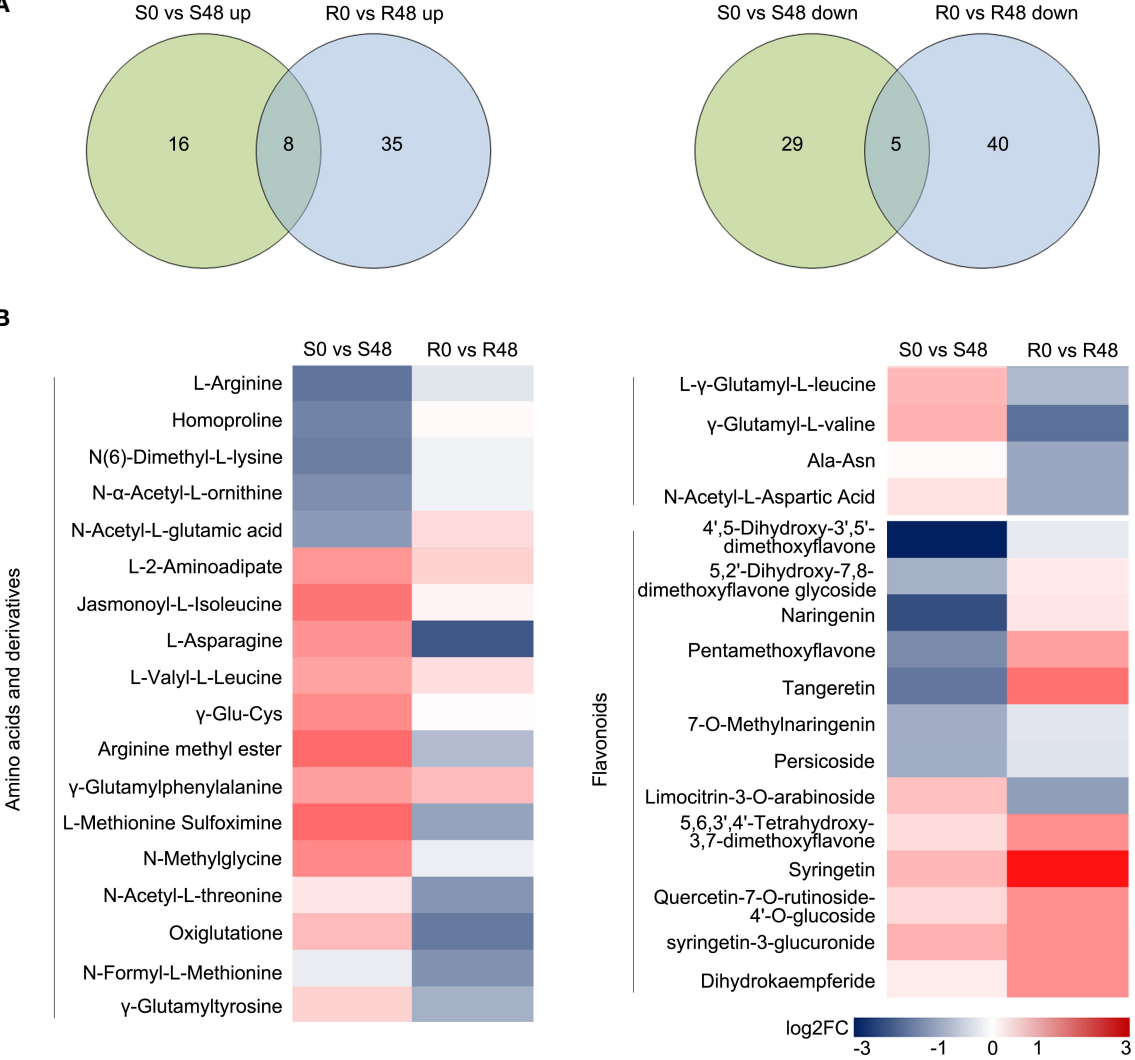
Unique DAMs in the two rice varieties after BPH feeding for 48 h. **(A)** Venn diagrams displayed the unique DAMs in two rice varieties before and after fed by brown planthopper. **(B)** Heat map showed the unique DAMs related to amino acids and derivatives and flavonoids in two comparison groups.

Venn diagrams were made to identify the unique and common DAMs in Nipponbare and BPH30T plants to clarify changes in metabolites. A total of 13 compounds were detected in both Nipponbare and BPH30T plants in the S0 vs S48 and R0 vs R48 comparison groups, including two amino acids and derivatives, one organic acid, four nucleotides and derivatives, four alkaloids, and two other compounds. In Nipponbare plants, 45 unique DAMs were identified, including 16 and 29 DAMs that were up-regulated and down-regulated, respectively. In BPH30T plants, 75 unique DAMs were identified, including 35 and 40 DAMs that were up-regulated and down-regulated, respectively (**Figure 6A**; **Supplementary Table 11**).

A heat map was made to characterize differences and patterns of variation in the 120 DAMs identified in Nipponbare and BPH30T plants. DAMs were identified from the following 11 classes: terpenoids, phenolic acids, flavonoids, nucleotides and derivatives, amino acids and derivatives, organic acids, lipids, others, lignans and coumarins, steroids, and alkaloids. Variation in primary metabolites, including nucleotides and derivatives, organic acids, and lipids, was similar among Nipponbare and BPH30T plants. But DAMs belonged the amino acids and derivatives class were down-regulated in BPH30T plants following BPH infestation. Variation in secondary metabolites such as phenolic acids, terpenoids, steroids, lignans, and coumarins and alkaloids, was not pronounced between Nipponbare and BPH30T plants. However, most DAMs in the flavonoid class showed an upward trend in *Bph30*-transgeneic plants, but down-regulated significantly in Nipponbare (**Figure 6B**; **Supplementary Figure 4**). These findings suggested that both amino acid and flavonoid metabolites might be involved in mediating the resistance to BPHs conferred by *Bph30*.

### Joint analysis of transcriptome and metabolome

**Figure 7:**
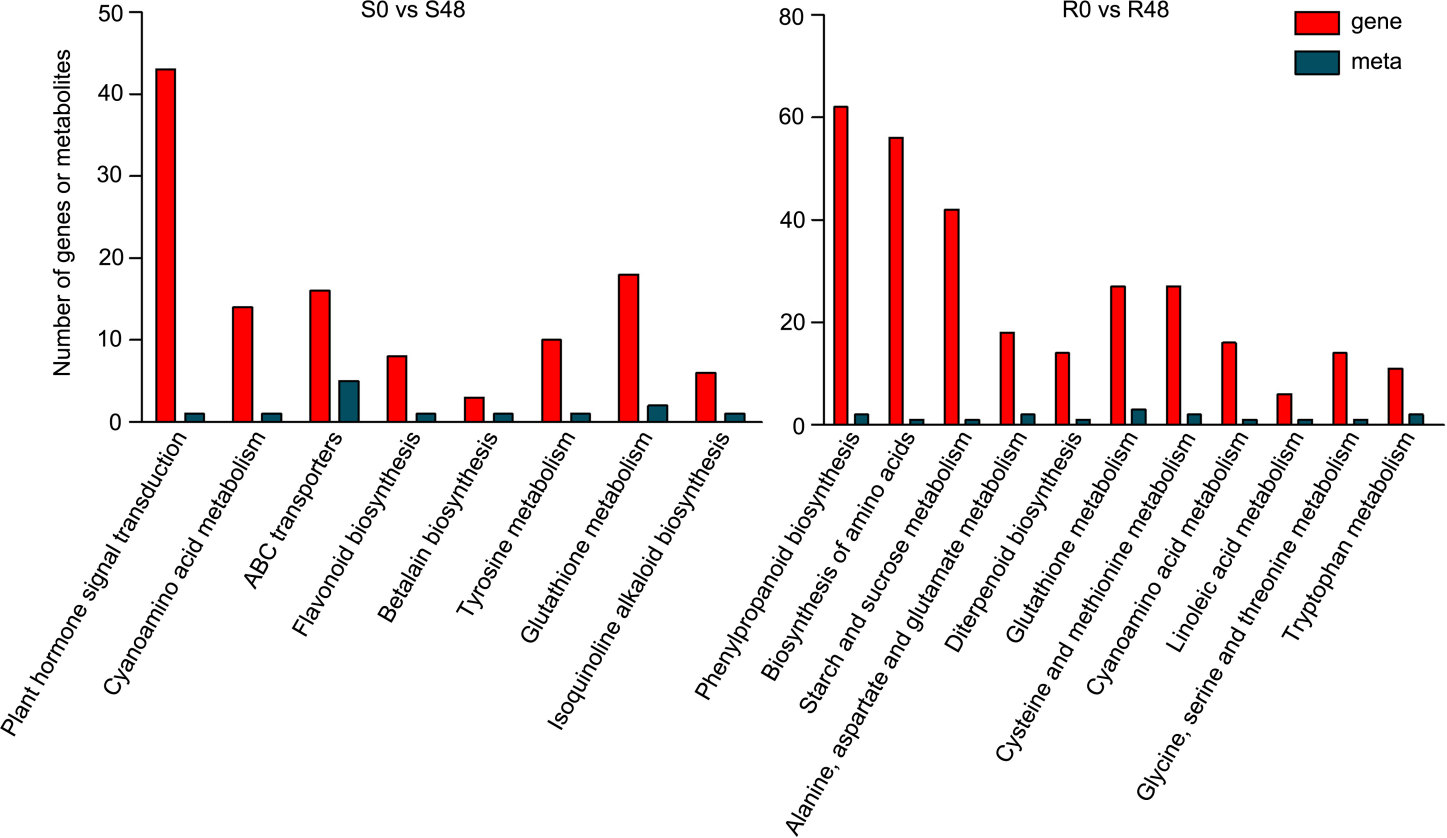
Transcriptomic and metabolomic joint analysis of Nipponbare and BPH30T after BPH infestation. KEGG enrichment analysis of the DEGs and DAMs detected from S0 vs S48 and R0 vs R48 groups.

To further characterize the control system of *Bph30* against brown planthopper feeding, we conducted a combined transcriptomic and metabolomic analysis of data from Nipponbare and BPH30T plants. KEGG functional analysis revealed some common functional pathways were enriched in by the two varieties. Such as, cyanoamino acid metabolism and glutathione metabolism, which DEGs and DAMs detected in the both varieties were enriched in. The pathways related in flavonoid biosynthesis, plant hormone signal transduction, ABC transporters, betalain biosynthesis, tyrosine metabolism and isoquinoline alkaloid biosynthesis were only enriched in Nipponbare, while, amino acid biosynthesis, phenylpropanoid biosynthesis, starch and sucrose metabolism, alanine, aspartate and glutamate metabolism, diterpenoid biosynthesis, cysteine and methionine metabolism, and so on, were specifically enriched in BPH30T plants. These findings showed that primary and secondary metabolic processes were activated in both varieties during BPH feeding, but the direction of metabolite transformation were significant different (**Figure 7**; **Supplementary Table 12**). The functional analysis also showed that the DEGs and DAMs detected in Nipponbare plants were mainly related to flavonoid biosynthesis and plant hormone signal transduction; by contrast, DEGs and DAMs in BPH30T plants were mainly enriched in amino acid biosynthesis and phenylpropanoid biosynthesis, suggesting those metabolic pathways mentioned above might play a vital role in mediating the BPH resistance conferred by *Bph30*.

**Figure 8:**
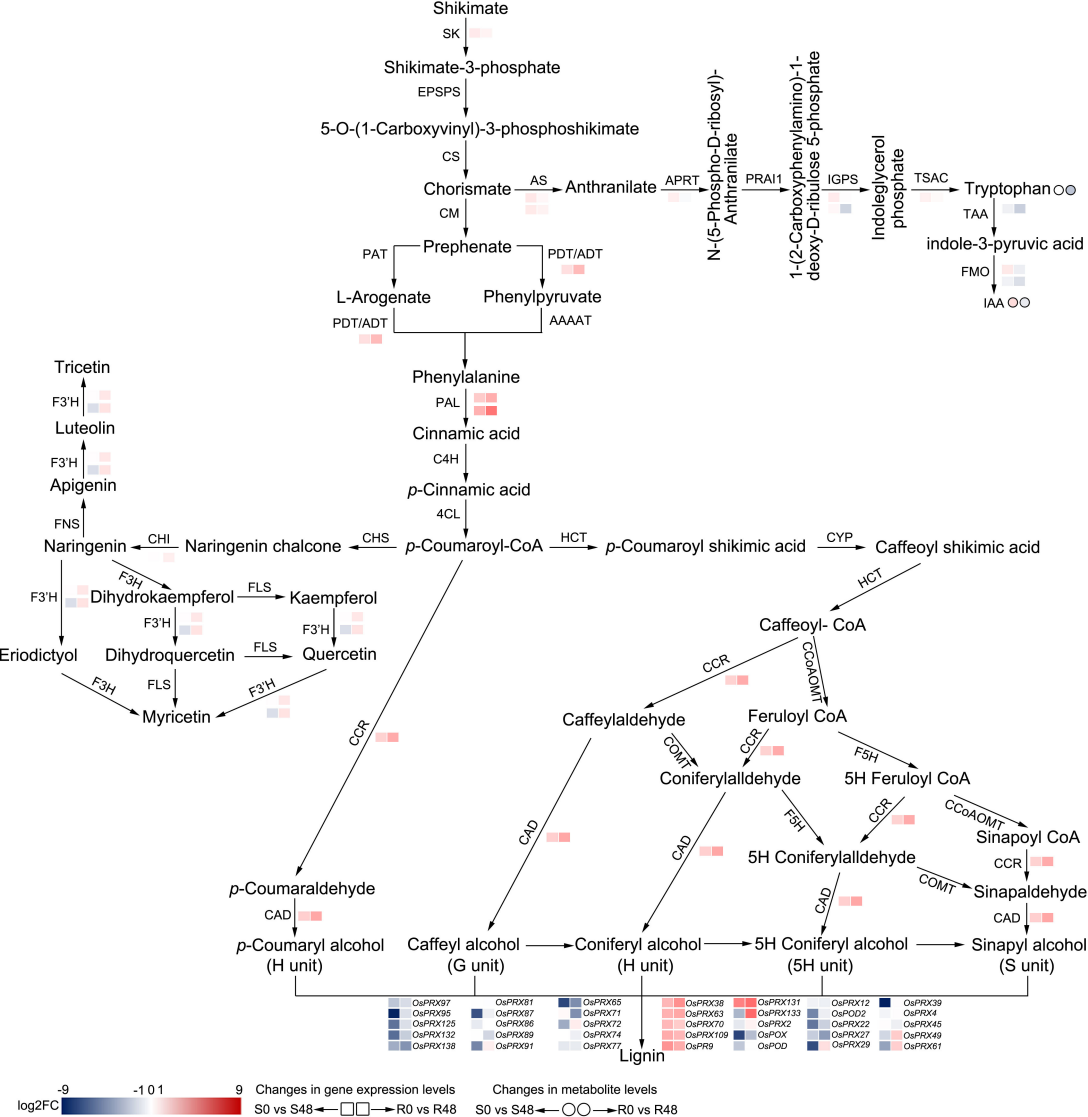
Reconstruction of the BPH-resistance related metabolism pathways with the DAMs and DEGs in Nipponbare and BPH30T fed by BPH for 48 h. S0 and S48 indicated Nipponbare fed by BPH for 0 h and 48 h, respectively. R0 and R48 indicated BPH30T fed by BPH for 0 h and 48 h, respectively. The rectangles represent the genes and the circles represent the metabolites. Color change represents the degree of variation.

The phenylpropanoid metabolism pathway was only enriched in BPH30T plants; this pathway plays key roles in plant growth and development, as well as the interactions between plant and environment (Dong and Lin, 2021). In this pathway, phenylalanine is converted to cinnamic acid by phenylalanine ammonia-lyase (PAL) firstly, then, cinnamic acid is converted to *p*-cinnamic acid by cinnamic acid 4-hydroxylase (C4H), finally, *p*-cinnamic acid is converted into *p*-coumaroyl-CoA by 4-coumarate-CoA ligase (4CL). Next, *p*-coumaroyl-CoA will enter two different metabolic pathways. One of the pathways is the flavonoids biosynthesis, the first step of this pathway is chalcone synthase (CHS) catalyze *p*-coumaroyl-CoA to naringenin chalcone. The other one is hydroxycinnamoyl-CoA shikimate/quinate hydroxycinnamoyl transferase (HCT) catalyze *p*-coumaroyl-CoA convert into *p*-coumaroyl shikimate, entering lignin biosynthetic pathway. In the phenylpropanoid metabolism pathway, up-regulation of one PAL gene (*Os02g0627100*) expression induced by brown planthopper feeding in BPH30T plants specifically. The expression of another PAL gene (*Os04g0518400*) was up-regulated in both Nipponbare and BPH30T plants, but the expression of this gene was up-regulated to a greater degree in the latter than in the former (**Figure 8**), indicating more initial compound (*p*-coumaroyl-CoA) were provided for the downstream secondary metabolic processes in *Bph30*-transgeneic plants after BPH infestation.

In the flavonoid biosynthesis pathway, the expression of one chalcone isomerase (CHI) gene (*Os12g0115700*) and two flavonoid 3’-hydroxylase (F3’H) genes (*Os03g0367101* and *Os10g0317900*) was down-regulated in Nipponbare plants; however, no changes in the expression of these genes were observed in BPH30T plants (**Figure 8**). Thus, we speculate that BPHs inhibit flavonoid synthesis, which increases the susceptibility of rice plants to infection when BPHs feed on rice plants lacking *Bph30*; the expression of *Bph30* maintains the normal synthesis of flavonoids under BPH infection in BPH30T plants, which impedes BPH infestation. In lignin biosynthesis, the expression of one cinnamyl alcohol dehydrogenase (CAD) gene (*Os04g0229100*) and one cinnamoyl-CoA reductase (CCR) gene (*Os02g0811600*) was up-regulated exclusively in BPH30T plants (**Figure 8**), suggesting the lignified cell wall plays a key role in mediating the BPH resistance conferred by *Bph30*; these findings are consistent with the results of our previous study (Shi et al., 2021).

Phenylalanine is one of the products from the shikimate pathway (Dong and Lin, 2021). In this pathway, shikimate is catalyzed by a series of enzymes to synthesize phenylalanine. We found the gene (*Os02g0749300*), which encoded shikimate kinase (SK) that catalyzes shikimate to shikimate 3-phosphate, was exclusively down-regulated in BPH30T plants only. The expression of *Os04g0406600*, which encodes the protein (arogenate/prephenate dehydratase, ADT/PDT) that catalyzes the conversion of prephenate to phenylpyruvate and arogenate to phenylalanine, was down-regulated in Nipponbare but up-regulated in BPH30T plants (**Figure 8**). These findings suggested that *Bph30* regulates the central flux of carbon from primary metabolism to secondary metabolism in BPH30T plants under BPH infection.

### Effects of BPH infestation on the IAA synthesis pathway

In rice, IAA is mainly synthesized from tryptophan (Trp). Chorismate, the intermediate product of shikimate pathway, is an initiator substrate for Trp synthesis. In the Trp synthesis pathway, we found that the expression of the anthranilate synthase (AS) genes *Os04g0463500* and *Os03g0826500*, the anthranilate phosphoribosyl transferase (APRT) gene *Os03g0126000*, and the Trp synthase alpha chain (TSAC) gene *Os07g0182100* was down-regulated exclusively in BPH30T plants. The expression of the indole-3-glycerol phosphate synthase (IGPS) gene *Os09g0255400* was down-regulated in both Nipponbare and BPH30T plants; however, the expression of this gene was down-regulated to a greater degree in BPH30T plants than that in Nipponbare; the IGPS gene *Os08g0320400* was specifically down-regulated in BPH30T plants (**Figure 8**). We also found the Trp content was significantly reduced in BPH30T plants following BPH infestation; however, BPH feeding activity had no effect on the Trp content in Nipponbare.

IAA is synthesized from Trp *via* tryptophan amino transferase (TAA) and flavonoid monooxygenase (FMO). First, Trp is converted to indole-3-pyruvic acid (IPA) by TAA. In the second step, IPA is converted to IAA by FMO (**Figure 8**). In the DEGs isolated from Nipponbare and BPH30T plants following BPH infestation, we found that the expression of *OsTAA1* (*Os01g0169800*), which encodes TAA, was down-regulated to a greater degree in BPH30T plants than in Nipponbare plants. The expression of *OsYUC5* (*Os12g0512000*), which encodes FMO, was down-regulated in BPH30T plants, and the down-regulation of the expression of *OsYUC9* (*Os01g0273800*) was more pronounced in BPH30T plants than in Nipponbare plants (**Figure 8**). Analysis of DAMs revealed that the content of IAA was lower in BPH30T plants following BPH feeding activity, but no change in the IAA content was observed in Nipponbare plants under the same conditions (**Figure 8**). The expression variation of the above genes was verified by qRT-PCR assay (**Supplementary Figure 5**). These findings indicate that IAA biosynthesis was inhibited by BPH infestation in BPH30T plants.

Overall, these findings suggested that *Bph30* can regulate the primary metabolic pathway (shikimate pathway and Trp biosynthesis), then affect the secondary metabolic process (phenylpropanoid metabolism, flavonoids and lignin biosynthesis) and IAA biosynthesis, exerting the function of resisting to brown planthopper. Noteworthy, the expression level of the genes that related IAA biosynthesis was lower in BPH30T plants than in Nipponbare and the contents of IAA were lower also in BPH30T plants than that in Nipponbare, suggesting that IAA might negatively regulate *Bph30*-mediated resistance to BPH in rice.

### Function of IAA in mediating BPH resistance conferred by *Bph30*

**Figure 9:**
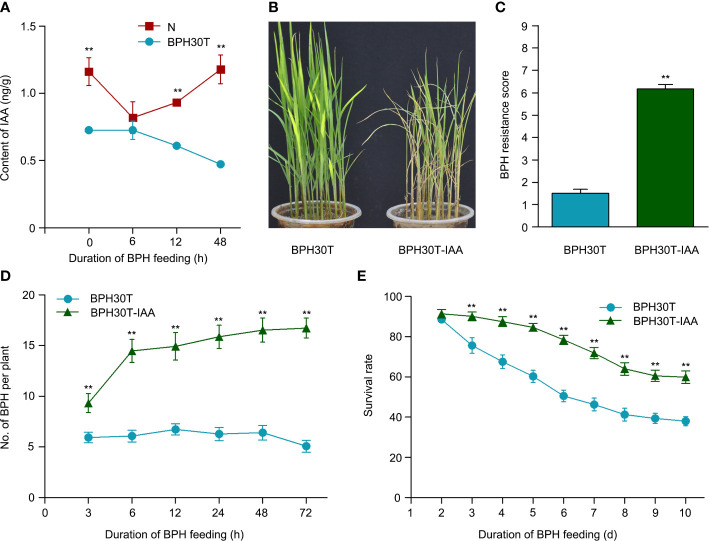
The IAA functioned in *Bph30*-mediated resistance to BPH. **(A)** The changes of IAA content in Nipponbare and BPH30T during BPH infestation. Data are means (three individual replicates) ± SEM. N, Nipponbare. **(B, C)** Seedling test identified the phenotype of resistance to BPH **(B)** and resistance score **(C)** of BPH30T treated with 1μm IAA (BPH30T-IAA). Data are means (at least 15 plants) ± SEM. **(D)** Selectivity of brown planthopper on BPH30T and BPH30T-IAA. **(E)** Survival rate of BPH feeding on BPH30T and BPH30T-IAA. In **(D, E)**, data represent the means ± SEM. Asterisks indicate significant differences (*represent *p* < 0.01).

The results of transcriptome, metabolome and combined analysis indicated IAA might play a vital role in *Bph30* against to BPH. To determine the role of IAA in mediating the BPH resistance conferred by *Bph30*, we measured the content of IAA in Nipponbare and BPH30T plants during BPH infestation. The content of IAA decreased significantly in BPH30T plants following BPH feeding activity; however, the content of IAA remained unchanged in Nipponbare plants under the same conditions (**Figure 9A**). The expression level of IAA-synthesis related genes was tested quantitatively also. The results of qRT-PCR showed the expression of *OsTAA1*, *OsYUC5*, and *OsYUC9* was significantly lower in BPH30T plants than in Nipponbare plants during BPH infestation (**Supplementary Figures 6A–C**).

Exogenous IAA was applied to BPH30T seedlings to clarify the role of IAA in mediating the BPH resistance conferred by *Bph30*. In bulk seedling test, the leaves of BPH30T plants treated with IAA were curled and yellow caused by BPH fed; by contrast, the leaves of control plants were normal (**Figures 9B, C**). The weight gain and honeydew excretion of BPHs were significantly higher when they fed on plants treated with IAA than when they fed on control plants (**Supplementary Figures 6D, E**). The host-choice tests were also performed, and the results showed that a significantly higher number of BPHs settled on the IAA treated plants than that settled on control plants from 3-72 h following the release of BPHs (**Figure 9D**). Furthermore, the BPH survival rate was significantly higher on IAA treated plants relative to control plants after 3 days (**Figure 9E**). These findings suggested that IAA negatively regulates BPH resistance conferred by *Bph30*.

## Discussion

In plants, resistance to biotic stress is thought to be determined by secondary metabolites. However, primary metabolites can also change when plants experience biotic stress. *Bipolaris oryzae* infection up-regulated the Trp pathway in rice leaves (Ishihara et al., 2008). *Magnaporthe grisea* infestation has been shown to alter the abundance of metabolites involved in the tricarboxylic acid (TCA) cycle and the synthesis of sugar alcohols and aromatic amino acids in barley, rice, and *Brachypodium distachyon* (Parker et al., 2009). BPH infestation induced changes in the abundances of primary metabolites in rice plants, such as amino acids, sugars, organic acids, lipids, and those metabolites involved in the Trp biosynthetic pathway, TCA cycle, shikimate pathway, GABA shunt, pentose phosphate pathway, wax biosynthesis, sterol biosynthetic pathway (Liu et al., 2010; Uawisetwathana et al., 2015; Peng et al., 2016; Zhang et al., 2018). Amino acids are essential nutrients that BPHs must obtain from the phloem sap of rice plants. When the resistant rice varieties were infected by BPH, the content of amino acids in phloem sap were significantly reduced, and the content of amino acid in BPH honeydew were significantly decreased, suggesting that the amounts of amino acids obtained by BPHs from resistant rice plants was reduced (Peng et al., 2017). We found that the amino acid biosynthesis pathway was down-regulated in BPH30T plants during BPH feeding (**Figure 3**). The expression of most genes involved in histidine, valine, leucine, isoleucine, serine, cysteine, alanine, glutamine, and homoserine biosynthesis was down-regulated in BPH30T plants but remained unchanged in Nipponbare plants following BPH infestation (**Supplementary Figure 7**). Thus, we speculate that *Bph30* might induce alterations in primary metabolites to mediate the response to invasion, or modify the composition of phloem sap; for example, *Bph30* reduces the content of amino acids, which makes the phloem sap a suboptimal food source for BPHs; this forces BPHs to colonize from *Bph30*-containing plants to susceptible plants to maximize nutrient acquisition.

Flavonoids play key roles in mediating the interaction between plants and parasites. A previous study showed that *Fusarium graminearum* infection increased the biosynthesis of flavonoid in wheat, and treatment of wheat spikes with exogenous kaempferide and apigenin enhanced the resistance of wheat to *F. graminearum* (Su et al., 2021). Vitexin and vitexin-2’’-O-arabinofuranoside are two flavonoid metabolites, which purified from *Basella alba*, significantly inhibit the growth rate of *S. litura* larvae when fed with these two substances (Aboshi et al., 2018). A previous study showed that tricin, which belongs to flavone, is common in gramineous plants, and can significantly improve the resistance of rice to brown planthopper. However, the content of tricin will significantly decrease in rice, when fed by brown planthoppers, indicated may be a protein in BPH regulates the tricin metabolize for feeding (Zhang et al., 2017). Current study showed BPH salivary protein 7 gene (*NlSP7*) was induced by tricin and NlSP7 regulated tricin metabolism in rice plants (Gong et al., 2022). Flavan-3-ols is a type of flavonoids with a high content in Tea (*Camellia sinensis*). Overexpression of Dihydro flavonol 4-reductase gene (*DFR*) and anthocyanidin reductase gene (*ANR*) in *Camellia sinensis* can significantly increase the content of flavan-3-ols and provide protection against feeding by *S. litura* (Kumar and Yadav, 2017). In sum, flavonoids are toxic and defensive secondary metabolites that protect plants from invaders; parasites can regulate the biosynthesis of flavonoids to promote their feeding activity. In our study, the flavonoid biosynthesis pathway was down-regulated exclusively in Nipponbare, and the flavonoid biosynthesis related genes in Nipponbare were also down-regulated (**Figure 3**; **Figure 8**), suggested that BPH infestation inhibited the synthesis of flavonoids and increased the susceptibility of rice plants to BPH; however, *Bph30* promoted flavonoid synthesis and increased the resistance of rice to BPHs.

The secondary cell walls of vascular plants are rich in lignin, which provides holding power for terrestrial plants and prevents the harm of invaders (Boerjan et al., 2003; Wang et al., 2017; Yang et al., 2017; Gallego-Giraldo et al., 2018). HCT catalyzes the conversion of *p*-coumarocyl CoA in the lignin biosynthesis. Reducing the expression of *HCT* in plants can reduce the lignin content and alter lignin composition in the second cell wall, and this can facilitate the cell wall degradation by cell wall-degrading enzymes secreted by invaders (Hoffmann et al., 2004). CmMYB19 is a transcription factor isolated from chrysanthemum. In overexpressing *CmMYB19-*transgenic chrysanthemums, the genes involved in lignin synthesis were up-regulated and the content of lignin was increased, which improved the resistance of chrysanthemum to attack by aphids (Wang et al., 2017). A recent report has shown that the BPH-resistance protein BPH6 interacted with the exocyst subunit OsEXO70H3 and SAMSL, and promotes the SAMSL secretion to apoplast, in where it increased lignin sedimentation in the cell wall, which promoted the rice plants resistance to planthoppers (Wu et al., 2022). The results of the above studies suggest that the cell wall can act as a physical barrier that prevents invaders from harming plants. Our previous study has shown that BPH30 enhances the hardness of the cell wall of the sclerenchyma and prevents the stylets of BPHs from impaling the sclerenchyma to reach the phloem for feeding (Shi et al., 2021). In this study, we found the CAD gene (*04g0229100*) and CCR gene (*02g0811600*), which are involved in lignin biosynthesis, were exclusively up-regulated expressed in BPH30T plants (**Figure 8**), indicating that lignin fulfilled a vital role in BPH30 resistance to brown planthopper, which may be contributed by the reinforcement of the physical barrier.

The biological function of phytohormone auxin is not only to regulate plant growth and development, but also to regulate plant stress resistance (Fu and Wang, 2011; Kidd et al., 2011; Qi et al., 2012; Gomes and Scortecci, 2021). In this study, we found the genes *OsTAA1*, *OsYUC5*, and *OsYUC9*, which related to IAA synthesis, were down-regulated expression in BPH30T plants, after BPH feeding, but unchanged in Nipponbare; the content of IAA in BPH30T plants was significantly lower than that in Nipponbare before and after BPH infestation. We also found IAA can reduce the resistance of *Bph30* to brown planthopper by the assay of exogenous application of IAA (**Figure 9**). Previous studies have demonstrated the auxin signal transduction is controlled by 3 switch proteins. The first switches are the auxin response factors (ARFs), which are transcription factors that activate the target genes of IAA. The second switches are the transcriptional repressors Aux/IAA, which bind to ARFs in the absence of auxin conditions, inhibiting IAA response genes expression. The third switches are the IAA receptors TIR1/AFB, which are the members of SCF^TIR1/AFB^ ubiquitination E3 complex. As the content of IAA increases, TIR1/AFB receptors bind to Aux/IAA repressor proteins, and mediates the degradation of Aux/IAA repressor proteins through ubiquitination pathway. Therefore, ARF proteins are released, which activates the expression of IAA response genes (Mockaitis and Estelle, 2008; Weijers and Wagner, 2016). Through analysis of DEGs that detected from the two rice varieties, we found there were seven and two Aux/IAA genes were down-regulated expression in Nipponbare and BPH30T plants, respectively; the expression of one TIR1/AFB gene was inhibited in BPH30T plants, but unchanged in Nipponbare. The ARF genes expression in both Nipponbare and BPH30T plants was inhibited after BPH feeding, but the down-regulated ARF genes in the two varieties differed (**Supplementary Figure 8**). These results indicated that IAA had a negative effect on BPH30 resistances to BPH, BPH30 altered IAA synthesis and regulated IAA signal transduction, thus improving the rice plants resist to brown planthopper. The target genes of IAA, which might increase susceptibility to BPH, remain unknown. In a future study, we plan to identify these genes.

BPH30 protein contains two LRDs that homologous to leucine-rich repeat domain (LRR) (Shi et al., 2021). Thus we speculated that LRDs may detected the proteins secreted into rice from BPH, transmitting the downstream signals that may involve in regulating endogenous auxin levels and the expression of auxin synthesis genes. But, the protein sequences of BPH30 in Nipponbare and BPH-resistant varieties AC-1613 were significantly different (Shi et al., 2021). So those differences in the protein sequences of BPH30 in Nipponbare from in AC-1613 may result in the loss of the function of recognition the BPH secreted proteins, leading to the failure of regulation of IAA content and biosynthesis genes expressions in rice during BPH infestation. Further researches are needed for the more detailed mechanisms.

## Conclusion

In summary, when attacked by BPH, the major BPH-resistance gene *Bph30* enhanced the flow of compounds to flavonoids and lignin biosynthesis through shikimate and phenylpropanoid metabolism pathways, and inhibited the biosynthesis of various amino acids and IAA, indicating BPH30 reprogrammed the direction of metabolite conversion. The results of this study demonstrate that IAA negatively regulates *Bph30*-mediated resistance to BPHs and that IAA signal transduction is involved in *Bph30*-mediated resistance to BPHs. Overall, our work systematically analyzed the molecular mechanism of *Bph30*-mediated resistance to brown planthopper in rice and provided theoretical guidance for rice breeding resistant to BPH.

## Data availability statement

All raw RNA sequencing data generated in this study have been deposited under the NCBI SRA database under BioProject PRJNA957551.

## Author contributions

LZ and AY conceived and supervised the project. KL (liukrice@163.com) revised the manuscript. SS designed the experiments and performed most of the experiments. XY, YW, SL, HX, PL, CL, KL (liukai11153@126.com), JC, GY, ZC, BW, and BLW performed some of the experiments. SS and WZ analyzed data and wrote the manuscript and contributed equally to this paper. All authors contributed to the article and approved the submitted version.
