# Supplementary material for: Integrated transcriptomics and metabolomics analysis provide insight into the resistance response of rice against brown planthopper
Source: Front Plant Sci. 2023 Jun 20;14:1213257. doi: 10.3389/fpls.2023.1213257 (PMC10327896; doi:10.3389/fpls.2023.1213257)
Supplement: Supplementary file 1 [file DataSheet_1.pdf]

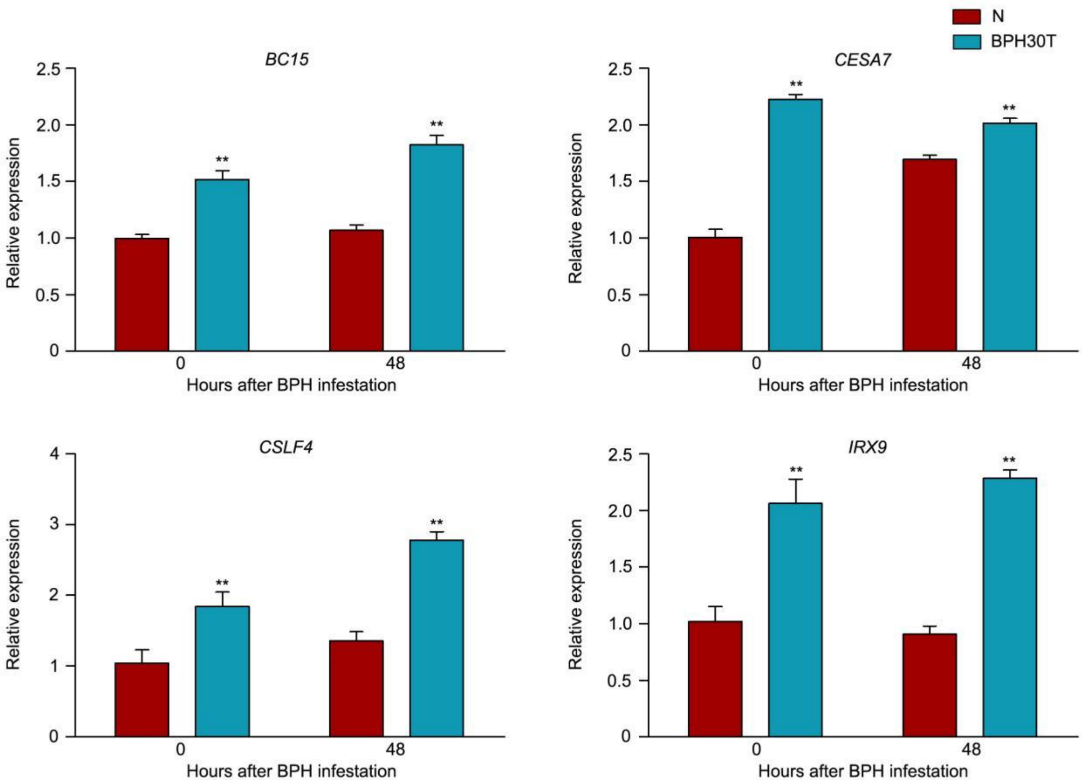

Supplementary Figure 1. The expression of cell-wall related genes in Nipponbare and BPH30T. *BC15*, *CESA7*, *CSLF4* involved in cellulose biosynthesis, *IRX9* involved in hemicellulose biosynthesis. Rice *OsAction1* was used as a reference control. Data represent means (three biological repeats) ± SD. Asterisks indicate significant differences revealed by one-way ANOVA (**$p < 0.01$). N, Nipponbare; BPH30T, *Bph30*-transgenic plants.

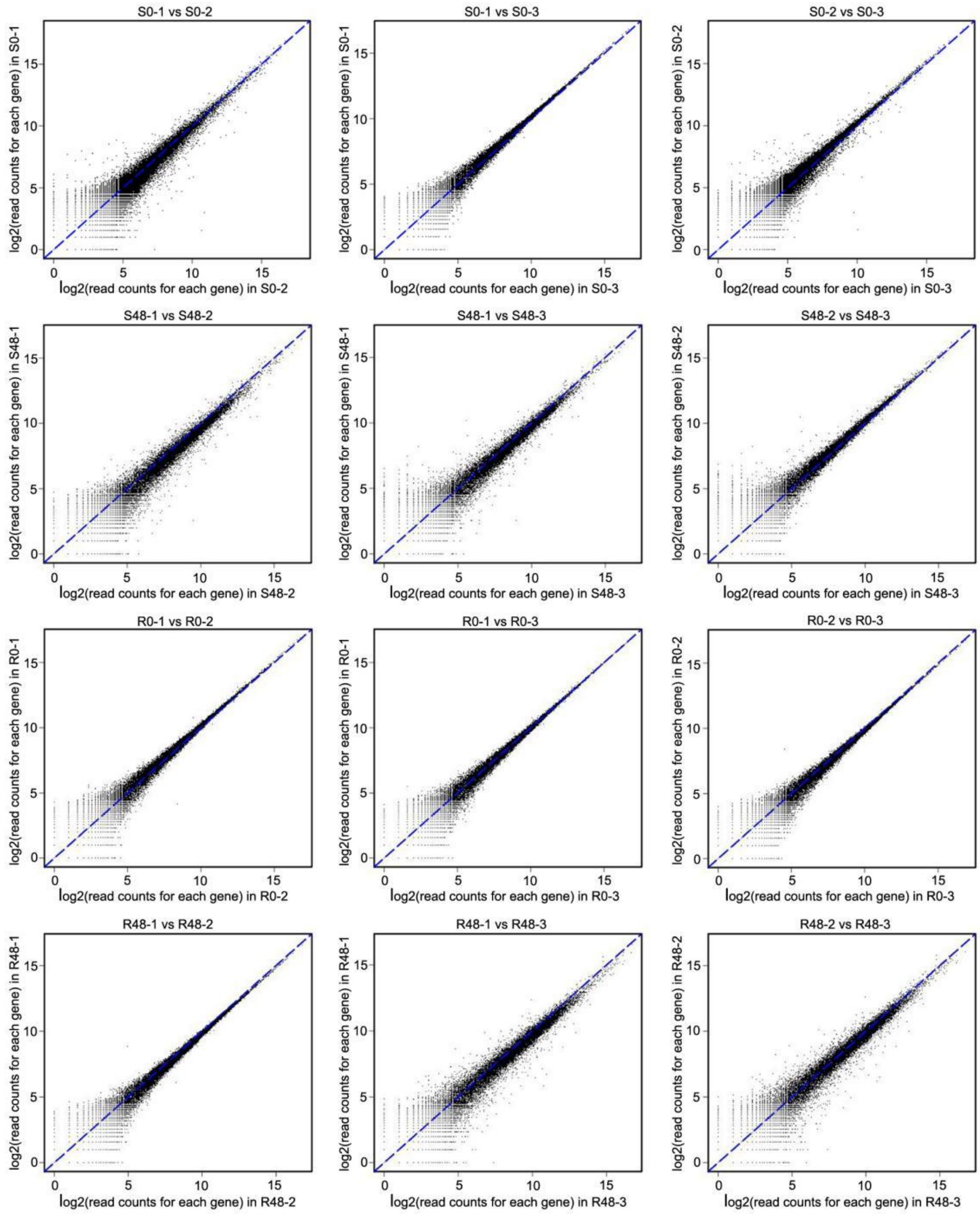

Supplementary Figure 2. The association between the replicates of the RNA-seq data of Nipponbare and BPH30T before and after BPH infestation. S, Nipponbare; R, BPH30T; 0, non-infested; 48, feeding for 48 h; 1, 2, 3 represent the three biological repeats.

A

Group	No. total DEGs	No. up-regulated DEGs	No. down-regulated DEGs
S0 vs S48	3407	912	2495
R0 vs R48	4313	1264	3049

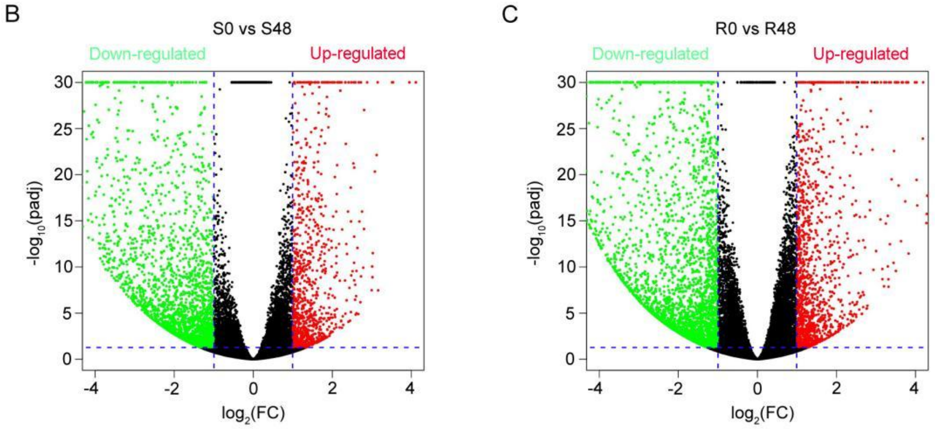

Supplementary Figure 3. Differentially expressed genes (DEGs) identified in Nipponbare and BPH30T fed by BPH for 48 h compared with unfed control. (A) The number of total, up- and down-regulated DEGs in two comparison groups. (B and C) Volcano plot illustrated the up- and down-regulated DEGs in two comparison groups S0 vs S48 (B) and R0 vs R48 (C). On the y-axis, the negative log10 adjusted corrected p values are plotted. On the x-axis, the log2 values of the fold changes shown in the transcriptome comparison.

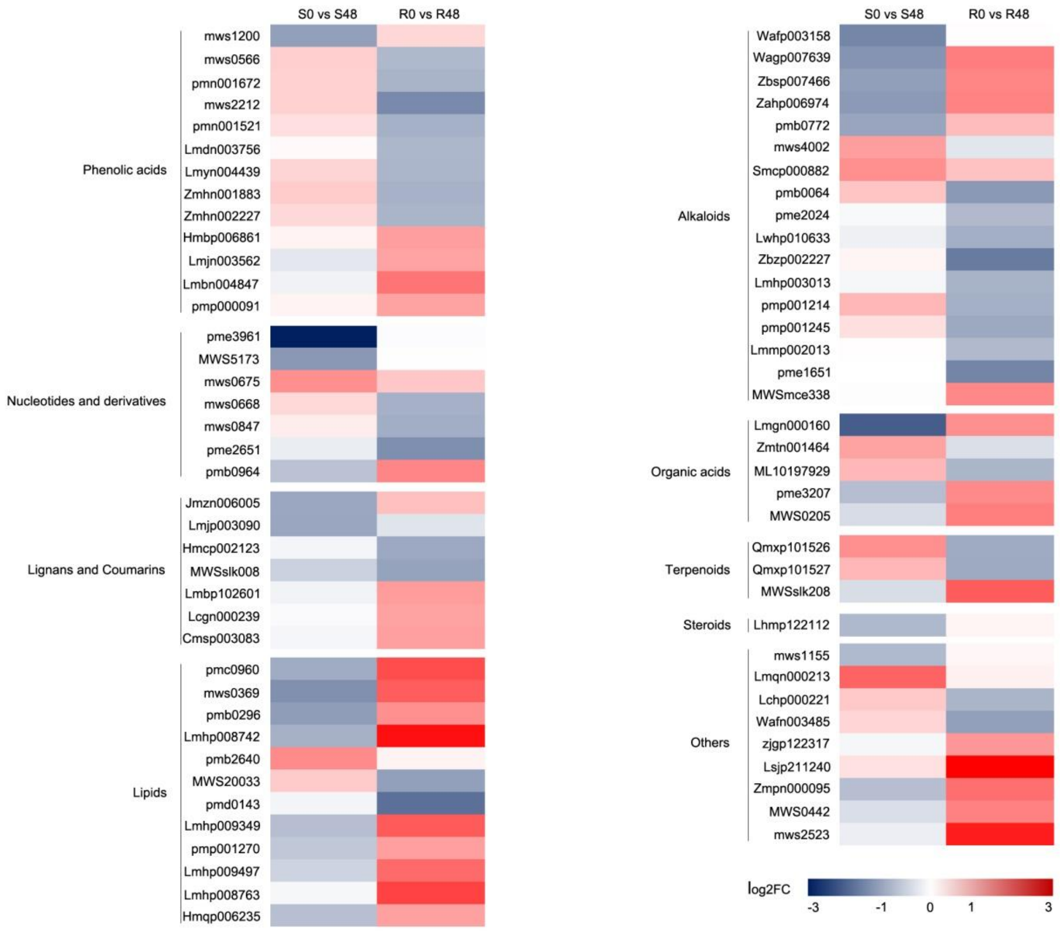

Supplementary Figure 4. Unique differentially accumulated metabolites (DAMs) in the two rice varieties after BPH feeding for 48 h. Heat map showed the unique DAMs related to phenolic acids, nucleotides and derivatives, lignans and coumarins, alkaloids, terpenoids, organic acids, steroids, lipids and others in two comparison groups.

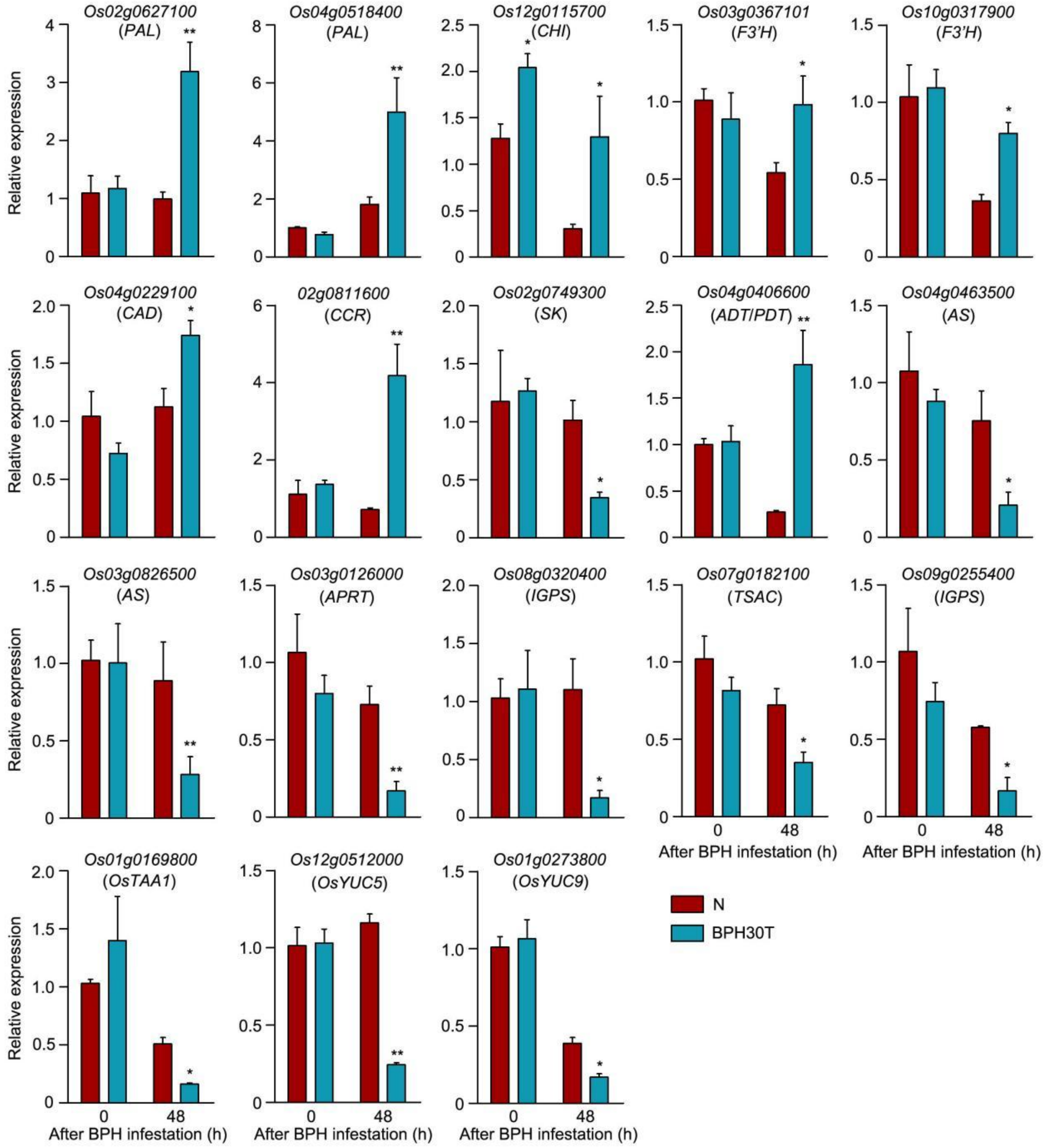

Supplementary Figure 5. The expression of genes that involved BPH-resistance related metabolism pathways in Nipponbare and BPH30T. Rice *OsAction1* was used as a reference control. Data represent means (three biological repeats) ± SD. Asterisks indicate significant differences revealed by one-way ANOVA (*$p < 0.05$ **$p < 0.01$). N, Nipponbare; BPH30T, *Bph30*-transgenic plants.

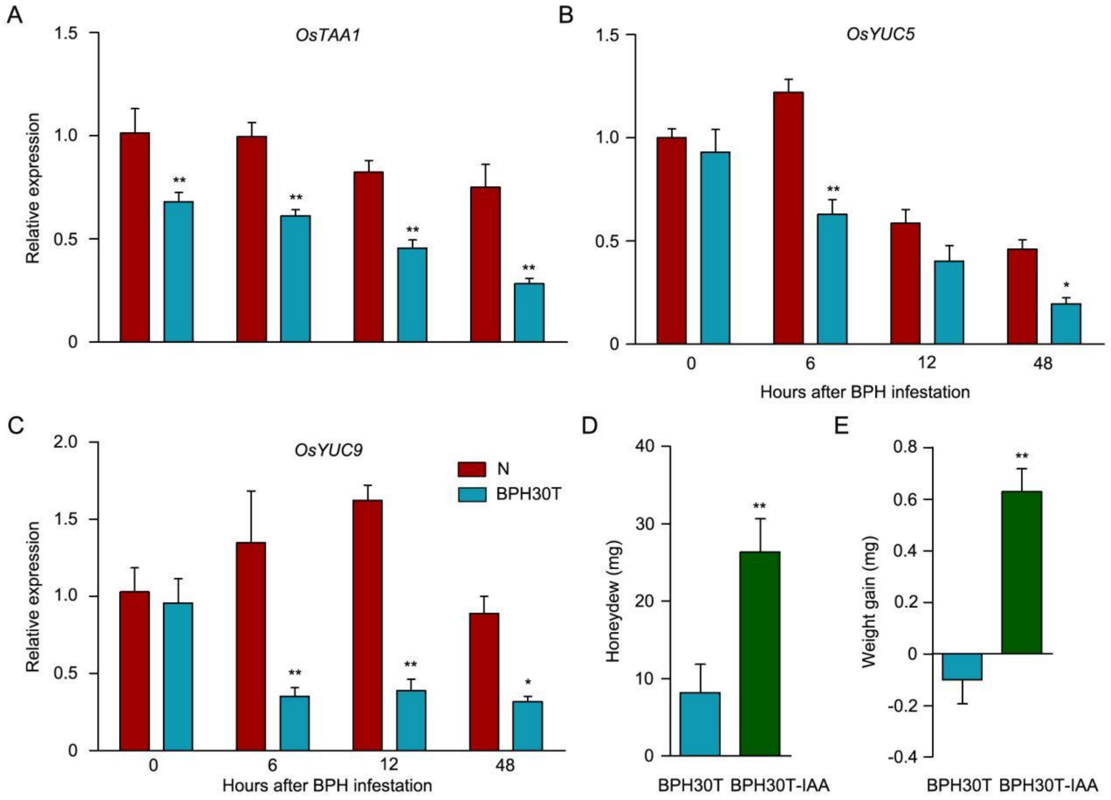

Supplementary Figure 6. IAA participates in *Bph30*-mediated resistance to BPH. (A-C) The expression of genes related IAA biosynthesis in Nipponbare and BPH30T during BPH infestation. Rice *OsAction1* was used as a reference control. Data represent means (three biological repeats) ± SD. Asterisks indicate significant differences revealed by one-way ANOVA (*p < 0.05 **p < 0.01). (D and E) Honeydew excretion (D) and weight gain (E) of BPH feeding on BPH30T and BPH30T-IAA for 2 days. Data represent the means (30 BPH insects) ± SEM, average values and SEM were calculated from three independent experiments, and 10 BPH insects were counted per experiment. N, Nipponbare; BPH30T, *Bph30*-transgenic plants; BPH30T-IAA, BPH30T treated with 1μm IAA.

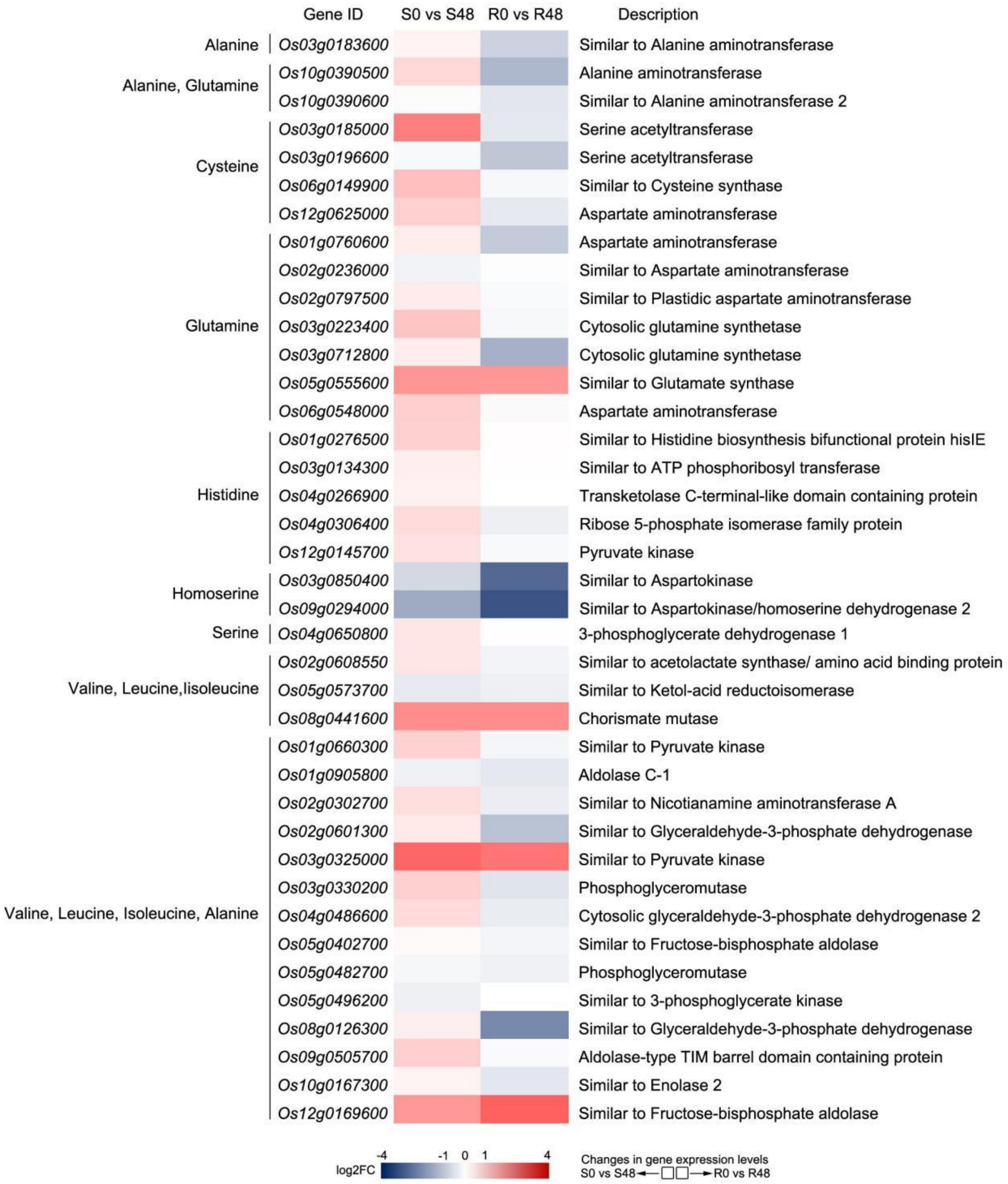

Supplementary Figure 7. The DEGs related amino acid biosynthesis in the two comparison groups. Heat map showed the unique DAMs related to histidine, valine, leucine, isoleucine, serine, cysteine, alanine, glutamine and homoserine biosynthesis in two rice varieties fed by BPH for 48 h compared with unfed control.

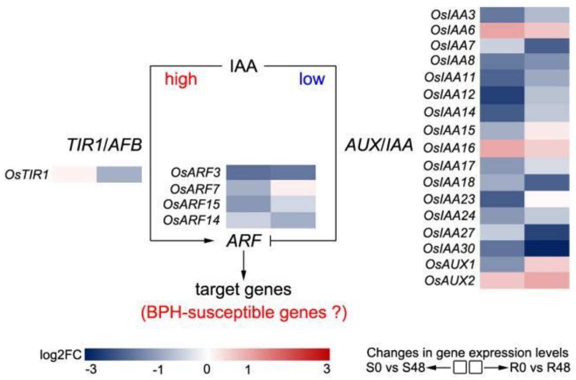

Supplementary Figure 8. DEGs in the two comparison groups comprising the IAA signal transduction pathway. The rectangles represent the genes, and color change represents the degree of variation.
